# Graph analysis based on SCN reveals novel neuroanatomical targets related to tinnitus distress

**DOI:** 10.3389/fnins.2024.1417032

**Published:** 2025-01-07

**Authors:** Yawen Lu, Yifeng Yang, Meijing Yan, Lianxi Sun, Caixia Fu, Jianwei Zhang, Yuehong Liu, Kefeng Li, Zhao Han, Guangwu Lin, Shihong Li

**Affiliations:** ^1^Department of Radiology, Huadong Hospital, Fudan University, Shanghai, China; ^2^Siemens Shenzhen Magnetic Resonance, Shenzhen, China; ^3^Department of Otolaryngology, Huadong Hospital, Fudan University, Shanghai, China; ^4^Pudong New Area People’s Hospital, Shanghai, China; ^5^Faculty of Applied Sciences, Macao Polytechnic University, Macau, China

**Keywords:** graph theoretic analysis, gray matter volumes, structural covariance network, tinnitus, magnetic resonance imaging

## Abstract

**Purpose:**

Tinnitus is considered a neurological disorder affecting both auditory and nonauditory networks. This study aimed to investigate the structural brain covariance network in tinnitus patients and analyze its altered topological properties.

**Materials:**

Fifty three primary tinnitus patients and 67 age- and sex-matched healthy controls (HCs) were included. Gray matter volume (GMV) of each participant was extracted using voxel-based morphometry, a group-level structural covariance network (SCN) was constructed based on the GMV of each participant, and graph theoretic analyses were performed using graph analysis toolbox (GAT). The differences in the topological properties of SCN between both groups were compared and analyzed.

**Results:**

Both groups exhibited small-world attributes. Compared with HCs, tinnitus patients had significantly higher characteristic path length, lambda, transitivity, and assortativity (*p* < 0.05), and significantly lower global efficiency (*p* < 0.05). Tinnitus patients had higher clustering coefficient and reduced gamma and modularity, but neither was remarkable. The hubs in tinnitus network focused on the temporal lobe. In addition, the tinnitus network was found to be reduced in robustness to targeted attacks compared with HCs. Besides, a significant negative correlation between Tinnitus Handicap Inventory (THI) score and GMV in the left angular gyrus (*r* = −0.283, *p* = 0.040) as well as left superior temporal pole (*r* = −0.282, *p* = 0.041) were identified.

**Conclusion:**

Tinnitus patients showed reduced small-world properties, altered hub nodes, and reduced ability to respond to targeted attacks in brain network. The GMV in the left angular gyrus and left superior temporal pole showed significant negative correlation with tinnitus distress (THI score), indicating potential therapeutic target.

## 1 Introduction

Tinnitus is a phantom sensation without external sound affecting 10–15% of adults and 32.0% of elderly individuals (Tunkel et al., 2014; Henry et al., 2020). It can be classified as subjective or objective, pulsatile or non-pulsatile, and primary or secondary according to clinical manifestations and etiology (Coelho et al., 2020). Ringing, buzzing, crickets, and hissing are common manifestations of tinnitus (Stouffer and Tyler, 1990; Coelho et al., 2020). The pathogenesis of primary tinnitus is still unclear. It was initially thought to be due to damage to the inner ear, but current studies suggest that the central nervous system plays a vital role in developing and maintaining tinnitus (Jastreboff, 1990; Takacs et al., 2017). Chronic tinnitus is often associated with a decline in cognitive functioning, especially attention, and a decline in emotional wellbeing, especially anxiety and depression (Baguley et al., 2013; Trevis et al., 2018). These symptoms indicate that the pathophysiology of tinnitus is not only a problem of the auditory network but also involves nonauditory perceptual networks including attention, emotion, and others (Leaver et al., 2016).

Recent studies have delved into the structural and functional networks of the brain using graph theoretic analyses (Fornito et al., 2013). These models define brain regions as nodes and links between them (anatomical or functional connections) as edges. Common tools for describing brain networks include resting-state functional magnetic resonance imaging (rs-fMRI) (for functional connectivity) and diffusion tensor imaging (DTI) (for white matter fiber bundle connectivity) (Iturria-Medina et al., 2007; Suo et al., 2018). Neuroimaging studies on tinnitus have revealed abnormal brain function alterations in both auditory and nonauditory systems. For instance, a previous study by our team used fMRI to detect changes in the intrinsic functional connectivity (FC) patterns between the dorsal attentional network (DAN) and other brain regions in tinnitus patients, and found that tinnitus patients not only altered the FC within the DAN, but also between the DAN and other brain regions (Hu et al., 2021). Lan et al. used rs-fMRI to construct functional brain networks for acute and chronic tinnitus, and demonstrated that prefrontal–limbic–subcortical regions exhibited obvious alterations in the topological properties of the brain networks during tinnitus chronicity (Lan et al., 2022). A study also assessed the relationship between white matter (WM) integrity and clinical variables in tinnitus patients using DTI, and found that WM integrity of the left auditory-limbic circuit in tinnitus is different in controls and DTI values correlated with depressive symptoms in tinnitus patients (Ryu et al., 2016).

Structural covariance networks (SCNs) serve as another non-invasive tool for exploring the microstructural abnormalities within the brain. They assist in calculating covariance relationships among structural indicators [e.g., gray matter volume (GMV), surface area, and cortical thickness] between brain regions within the same group of participants. Current opinion suggests that SCNs may reflect synchronous development between various brain regions (Alexander-Bloch et al., 2013b). Studies have demonstrated a strong correspondence between SCNs and rs-fMRI as well as DTI (Chen et al., 2022; Lee et al., 2023). Applications of SCN-based studies have been extended to a range of neurological disorders, including Parkinson’s disease, Alzheimer’s disease, depression, and others (Zhou et al., 2020; Dai et al., 2023; Rohrsetzer et al., 2023). Previously, several neuroimaging studies of tinnitus have demonstrated abnormal alterations in brain structural properties that include both auditory and non-auditory systems (Lin et al., 2020; Wei et al., 2020; Lan et al., 2022). A study that combined cortical thickness and subcortical volume confirmed alterations in the topology of the tinnitus brain network (Lin et al., 2020). Another study, which investigated structural changes in tinnitus patients’ brain networks before and after 6 months of sound treatment, demonstrated a trend toward recovery after treatment (Wei et al., 2020). However, while these studies revealed alterations in the structural covariate network and post-treatment plasticity in tinnitus patients, further exploration of these alterations is crucial to unraveling the mechanisms behind tinnitus pain and identifying potential therapeutic targets.

In our study, we constructed SCNs based on GMV and investigated alterations in the topological properties of a large-scale structural brain network in patients with primary chronic tinnitus. Furthermore, we studied the correlation between these changes and tinnitus distress scores.

## 2 Materials

### 2.1 Participants

We recruited patients with primary tinnitus who visited Huadong Hospital during the period between July 2020 and August 2023, as well as sex- and age-matched healthy controls (HCs). The inclusion criteria for tinnitus patients were (1) age between 14 to 80 years old, (2) persistent tinnitus for more than 3 months, and (3) right-handedness. Participants were excluded if they met any of the following criteria: (1) tinnitus caused by vascular conditions, otitis, or other organic brain lesions; (2) other systemic diseases that not under reasonable control; (3) contraindications to MRI; or (4) a family history of mental illness or inherited neurological disorders. Written informed consent was obtained from all participants. The study was approved by ethical committee of Huadong Hospital (2020 K135).

### 2.2 Clinical data collection of patients with tinnitus

The tinnitus patients recruited in this study were recorded with their sex, age, and duration of tinnitus. In addition, the Tinnitus Handicap Inventory (THI) was used to quantify the impact of tinnitus on daily life (Newman et al., 1996). The THI score ranges from 0 to 100, indicating the severity of tinnitus. Furthermore, pure tone audiometric data were collected from tinnitus patients, including the hearing thresholds measured at 125, 250, 500, 1,000, 2,000, 3,000, 4,000 and 8,000 Hz in each ear. The pure tone average (PTA) in the tinnitus side ear was determined as the average thresholds across 500, 1,000, 2,000 and 4,000 Hz. If tinnitus is bilateral, the average hearing in both ears is measured.

### 2.3 MRI acquisition

All patients underwent MRI using a 3.0 T system (MAGNETOM Prisma, Siemens Healthcare, Erlangen, Germany) with a 20-channel head coil. We used foam padding to reduce motion artifacts and earplugs to minimize noise. Participants were asked to remain still during the scan. Routine MRI sequences including T2 weighted imaging (T2WI), T1 weighted imaging (T1WI), and Diffusion Weighted Imaging (DWI) were used to exclude other lesions in the brain. The MRI acquisition protocols are described in Supplementary Table S1.

Additional 3D-T1-weighted images were acquired of all participants. The specific scanning parameters are as follows: repetition time (repetition time, TR) = 2,300 ms, echo time (echo time, TE) = 2.32 ms, inversion time (inversion time, TI) = 900 ms, flip angle = 8°, number of layers = 192 layers, layer thickness = 0.9 mm, continuous scanning with no interval, matrix = 256 × 256, acquisition time = 5min21s.

### 2.4 MRI preprocessing

Data preprocessing and voxel-based morphometry (VBM) analyses were conducted using the Statistical Parametric Mapping (SPM12) toolbox based on the MATLAB platform. The preprocessing included the following 5 steps: (1) data conversion: converting all MR images from DICOM format into NIFTI format; (2) bias field correction: reducing the difference in luminance values of the same tissue, facilitating tissue segmentation; (3) segmentation: segmenting format-converted T1 images into gray matter, white matter, and cerebrospinal fluid based on the DARTEL algorithm; (4) spatial normalization: aligning the gray matter images to standard MNI space; and (5) modulation: applying the deformation field generated during spatial normalization to the segmented gray matter image to correct the bias generated during the process. VBM analysis required the modulated image to be first smoothed with an 8-mm smoothing kernel. Voxels with gray values <0.15 in gray matter images were further excluded from statistical analysis to eliminate edge effects in gray and white matters.

### 2.5 Construction of SCNs and graph theoretic analysis

#### 2.5.1 Construction of SCNs

**Figure 1:**
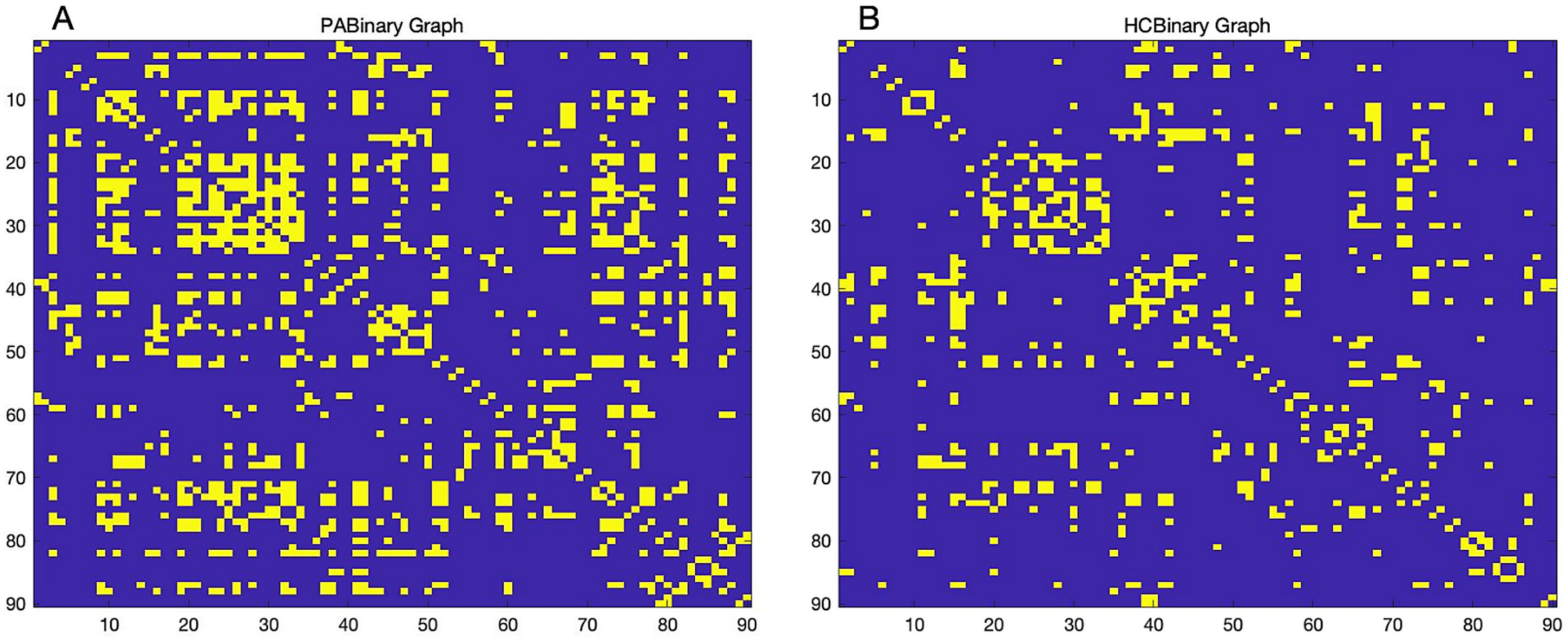
Binary adjacency matrix. Binary adjacency matrix of patients with tinnitus **(A)** and healthy controls **(B)** at a minimum network density of 0.16. Yellow color indicates the presence of connections, and blue color indicates the absence of connections. HC, Healthy control; PA, patients with tinnitus.

We used automated anatomical labeling (AAL) brain mapping to divide the modulated gray matter images into 90 brain regions. By correcting for participants’ sex, age, and total intracranial volume as covariates, Pearson correlation coefficients were then calculated between GMVs of all brain regions in each group, which ultimately resulted in a 90 × 90 correlation matrix, forming the group-level SCN. A threshold was determined based on the specific network sparsity, and the association matrix was converted into a binary matrix with values of 1 or 0 for further analysis (Figure 1).

#### 2.5.2 Network sparsity setting

Network sparsity refers to the ratio of the number of edges present in the network to the maximum possible number of edges. The formula for calculating it is [*D* = *E*/ (*N* (*N* – 1))/2], where *E* represents the actual existence of connected edges and *N* represents the number of nodes. In this study, the network sparsity threshold was set within a certain network density range (*D*_min_ = 0.16, *D*_max_ = 0.50, step = 0.02). The *D*_min_ denoted the minimum density that enables the network to be fully connected, meaning that the minimum network sparsity needed to ensure that both SCNs were fully connected without any isolated nodes. The minimum sparsity was automatically determined using graph-theoretical analysis toolbox (GAT), and the value was 0.16. *D*_max_ was set to 0.50 because brain networks were generally considered to be highly stochastic when the network density was greater than 0.5 and were usually considered to be abiotic (Hosseini et al., 2012).

#### 2.5.3 Measurement of brain network graph theory parameters

Local and global network parameters were evaluated over a range of network densities (0.16–0.50) with a step size of 0.02. The global network measures included clustering coefficient (Cp), normalized clustering coefficient (gamma, 𝛄), characteristic path length (Lp), normalized characteristic path length (lambda, 𝛌), small-world properties (sigma, 𝛔), and global efficiency (Eg). The parameter 𝛔 was used to measure the small-world properties of the network. The brain network was considered to have small-world properties when 𝛄 > 1 and 𝛌 ≈ 1 or 𝛔 > 1 (Rubinov and Sporns, 2010). Additionally, the assortativity, transitivity, and modularity of both groups were also described and compared. The local network measures included node degree and betweenness, which were defined as the number of nodes connected to the node and the number of shortest paths passing through the node, respectively. To estimate group differences in network indicators, we used functional data analysis (FDA) and area under the curve (AUC) analyses.

The hubs evaluated in the network using *D*_min_ as the threshold value. If the betweenness centrality of a node exceeded 2 standard deviations (SD) above the mean betweenness centrality in the network, it was considered to be a hub node (Mak et al., 2016).

The nodes of the network were subject to targeted attacks and random failures, and the changes in the relative size of the network were analyzed to assess the ability of the tinnitus brain network to respond to damage. Targeted attack involved the sequential removal of nodes from the highest to the lowest node degree while random failure referred to random removal of nodes until all 90 nodes were removed and repeated 1,000 times to ensure the stability of the results. The node degree could be used to identify nodes that had a remarkable impact on the network structure during removal (Iyer et al., 2013). The relative size of the network represented the ratio of the total number of edges of the subnetwork after each node removal to the total number of edges of the SCN. Subsequently, the degree of network robustness was evaluated by calculating AUC.

### 2.6 Statistical analysis

Statistical analyses were performed using the SPSS software (v.26.0). The Shapiro–Wilk test was used to assess whether the variables were normally distributed. Categorical data were compared using the chi-square test for between-group comparisons. Brain networks were compared between two groups of participants using GAT and a nonparametric permutation test with 1,000 repetitions in each group was used to test whether between-group differences in global and local network measures were statistically significant. The meaningful brain regions in the tinnitus group in the nodal analysis were set as regions of interest (ROI), and biased correlation analyses were performed between their GMV values and THI scores, with covariates of gender, age, and total intracranial volume (TIV). A 2-tailed *p* < 0.05 was considered statistically significant.

## 3 Results

### 3.1 Demographic and clinical data

A total of 53 patients with chronic primary tinnitus (median age 43.2 years, 20 females) and 67 age- and sex-matched HCs (median age 42.0 years, 32 females) were included (Table 1). There was no statistically significant difference in age and sex between the two groups. Patients with tinnitus had a median tinnitus duration of 50 months and a median THI score of 48. Thirty-six patients had audiometric testing results, and the average hearing level of tinnitus-affected side ear was 42.83 ± 25.44 decibels dB HL (range: 8.75–115.00 dB HL).

**Table 1:** Demographic information of the participants.

	PAs (*n* = 53)	HCs (*n* = 67)	*p*-value
Median age (year)	43.2 (31.0–54.5)	42.0 (29.0–53.0)	0.638
Sex (female/male)	20/33	32/35	0.271
Duration of tinnitus (months)	50 (6.5–42)	NA	NA
THI score	48 (26–68)	NA	NA

NA, not applicable; HCs, healthy controls; PAs, patients with tinnitus; THI, tinnitus handicap inventory.

### 3.2 Global network measurements

#### 3.2.1 Between-group differences in global network measures

**Figure 2:**
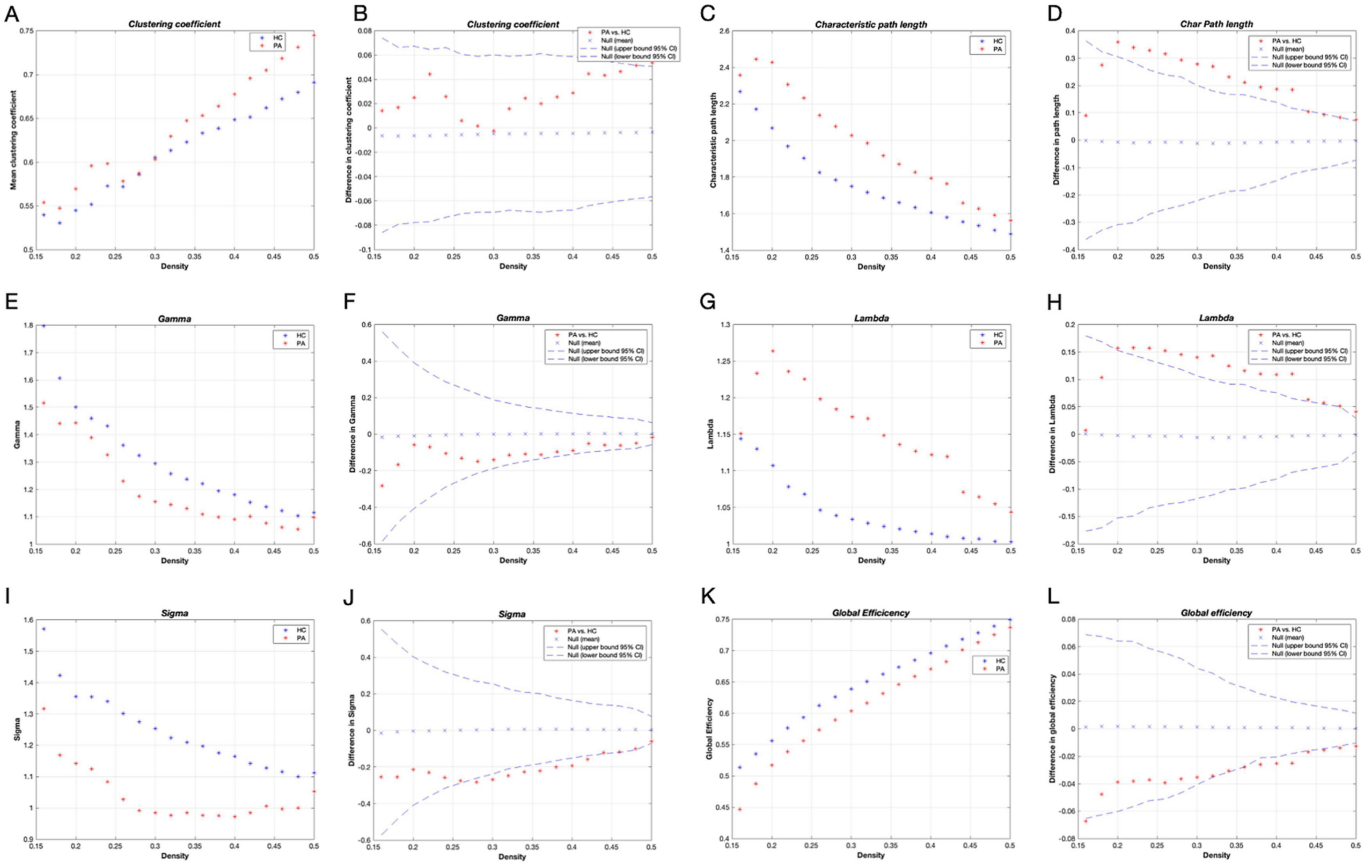
Global network metrics in different network density and intergroup differences. Horizontal coordinates indicate network sparsity; dashed lines indicate 95% confidence intervals, and red stars outside the 95% confidence intervals indicate statistically significant differences between groups (*p* < 0.05), with positive values indicating that the PA > HC and negative values indicating that the PA < HC; Clustering coefficient **(A,B)**, Characteristic path length **(C,D)**, Gamma **(E,F)**, Lambda **(G,H)**, Sigma **(I,J)**, and Global efficiency **(K,L)** of the tinnitus patients and HCs. HC, healthy control; PA, patients with tinnitus.

The variations in global network measurements with network density in patients with tinnitus and HCs are illustrated in Figure 2. Both groups had small-world properties (𝛔 > 1); patients with tinnitus demonstrated smaller small-world properties compered to HCs within the network density range of 0.28–0.42 (*p* < 0.05) (Figures 2I,J). Furthermore, within the network density range of 0.20–0.50, patients with tinnitus exhibited considerably higher Lp and 𝛌 compared with HCs (*p* < 0.05) (Figures 2G,H). Lastly, Eg was substantially lower in patients with tinnitus than in HCs in the network density range of 0.38–0.42 (*p* < 0.05) (Figures 2K,L).

Transitivity and assortativity were remarkably higher in patients with tinnitus over most of the range of network densities (*p* < 0.05) (Supplementary Figure S1). Cp was higher while 𝛄 and modularity were lower in patients with tinnitus compared with HCs, but neither was considerable (*p* > 0.05).

#### 3.2.2 FDA and AUC analysis of global network measurements

We compared the intergroup differences in AUC between patients with tinnitus and HCs over a range of network densities. Similar to the observed differences observed in density, the tinnitus network had significantly higher levels of transitivity (*p* = 0.022), assortativity (*p* = 0.001), and Lp (*p* = 0.005). Patients with tinnitus demonstrated less small-worldness compared with HCs (*p* = 0.066). Furthermore, the results from FDA were consistent with the AUC results, indicating that the tinnitus network had high transitivity (*p* = 0.023), assortativity (*p* = 0.001), and Lp (*p* = 0.005).

### 3.3 Local network measurements

**Figure 3:**
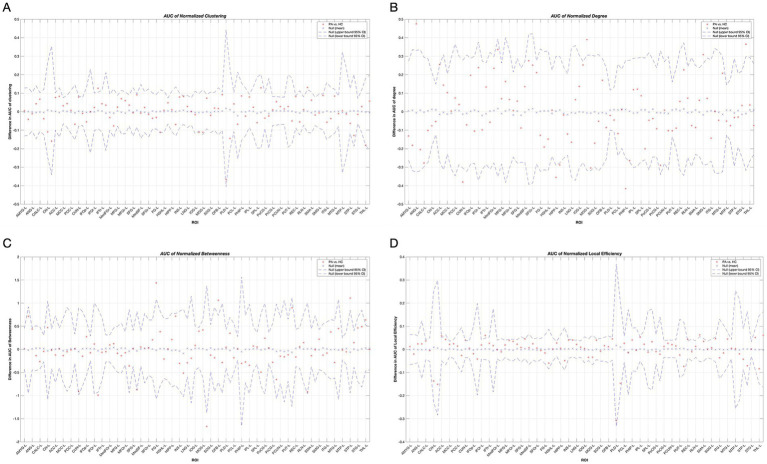
Between-group differences in normalized clustering coefficients **(A)**, degree **(B)**, node betweenness **(C)** and local efficiency **(D)** in tinnitus group and HCs over a range of network densities. Red stars indicate differences between both groups. All regions survived after FDR correction. Abbreviations for each brain region in the supplementary data. HC, Healthy control; PA, patients with tinnitus.

As shown in Table 2 and Figure 3, patients with tinnitus had noticeably larger Cp in the right infraorbital subfrontal gyrus (IFOr) and right rolandic operculum (RLN), but lower Cp in the right inferior occipital gyrus (IOG).

**Table 2:** Differences in AUC of local network measurements between both groups.

Local property	PA < HC	*P*-value	PA > HC	*p*-value
Clustering coefficient				
	IOG-R	0.035	IFOr-R RLN-R	0.007^**^ 0.023
Degree				
	CUN-L HIPP-L HIPP-R PHIP-L	0.016 0.012 0.047 0.001^**^	ANG-L ACC-L MOG-L SMG-L STG-R	0.002^**^ 0.043 0.008^**^ 0.014 0.023
Node betweenness				
	IFOr-R MOG-L	0.031 0.017	FG-L OFB-L STP-L THL-L	0.017 0.006^**^ 0.016 0.026
Local efficiency				
	/	/	ACC-L CUN-L IFOr-R SFG-R INS-R REC-L RLN-R	0.034 0.044 0.007^**^ 0.045 0.045 0.015 0.029

**p < 0.01. Abbreviations for each brain region in the Supplementary data. HC, Healthy control; PA, patients with tinnitus.

The degrees of the left cuneus (CUN), bilateral hippocampus (HIPP), and left parahippocampal gyrus (PHIP) in patients with tinnitus were significantly lower than those in HCs. Conversely, the degrees of the left angular gyrus (ANG), left anterior cingulate gyrus (ACC), left middle occipital gyrus (MOG), left supramarginal gyrus (SMG), and right superior temporal gyrus (STG) were noticeably higher in patients with tinnitus than that in HCs.

In the tinnitus network, the right IFOr and left MOG had significantly lower node betweenness, whereas the left fusiform gyrus (FG), left olfactory cortex (OFB), left superior temporal pole (STP), and thalamus (THL) had significantly higher node betweenness.

Furthermore, the local efficiency of the left ACC, left CUN, right IFOr, right superior frontal gyrus (SFG), right insula (INS), left rectus gyrus (REC), and right RLN was significantly higher in patients with tinnitus compared to HCs.

All the aforementioned regions survived after false discovery rate (FDR) correction (*p* < 0.05).

### 3.4 Degree distribution

**Figure 4:**
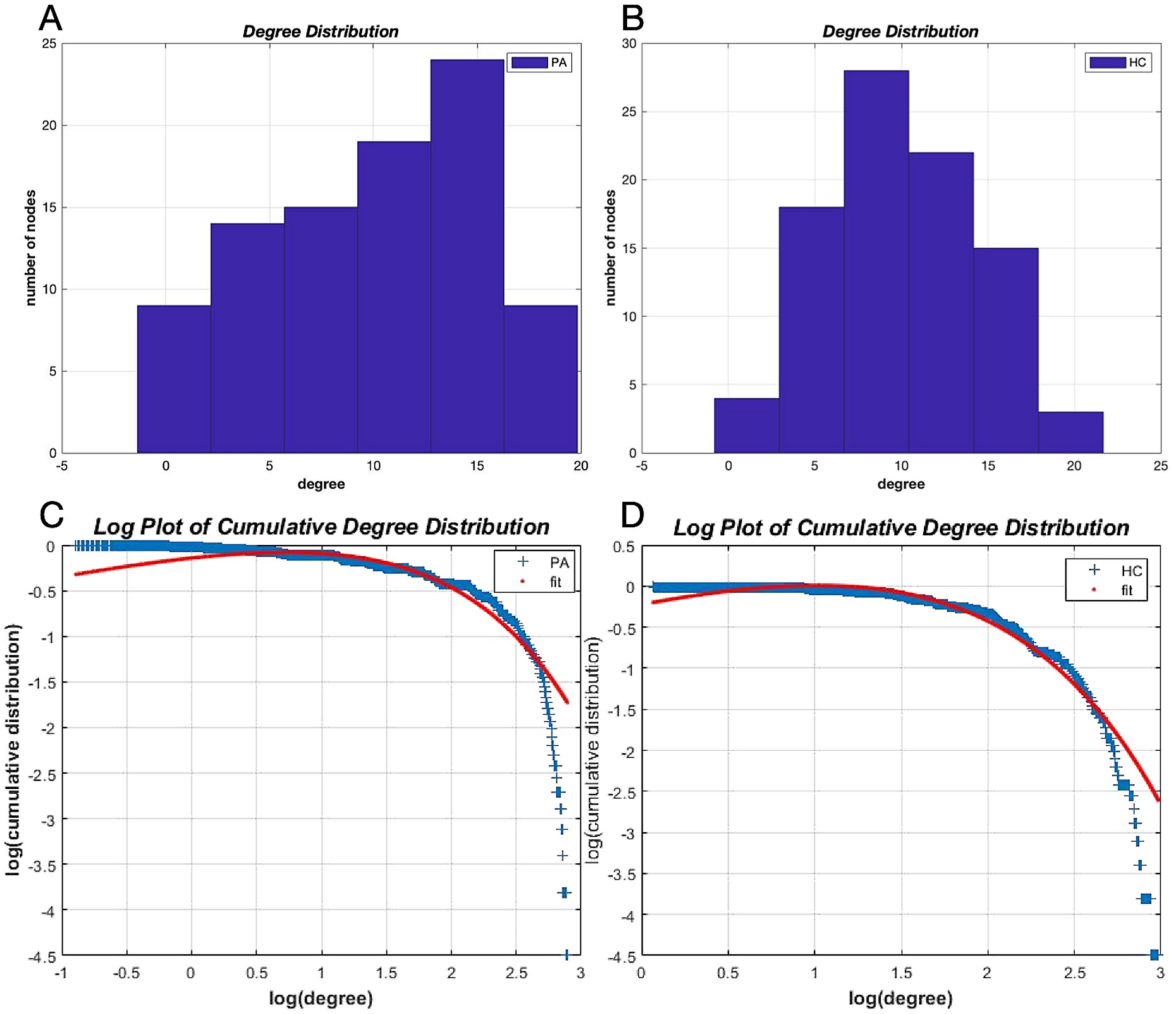
Degree distributions. Degree distributions for patients with tinnitus **(A)** and HCs **(B)** and logarithmic plots of the cumulative degree distributions with AUC as the threshold **(C,D)**. The blue line indicates the network cumulative degree distribution. The red line represents the exponentially truncated power-law curve fitting the network cumulative degree distribution. AUC, Area under the curve; HC, healthy control; PA: tinnitus patients.

The network degree distributions of both groups followed an exponentially truncated power-law distribution (Figures 4C,D). The exponential estimate for HCs was 1.62, while for patients with tinnitus it was 1.29, and the truncation degree for HCs was 4.44 versus 6.99 for patients with tinnitus. The *R*-squared value of the fitted distributions in the two groups was 0.98 for HCs and 0.93 for tinnitus patients, respectively. The degree distributions in tinnitus patients were non-normal, indicating a disorder (Figure 4A), whereas the degree distributions in HCs were relatively normal (Figure 4B).

### 3.5 Network hub identification

**Figure 5:**
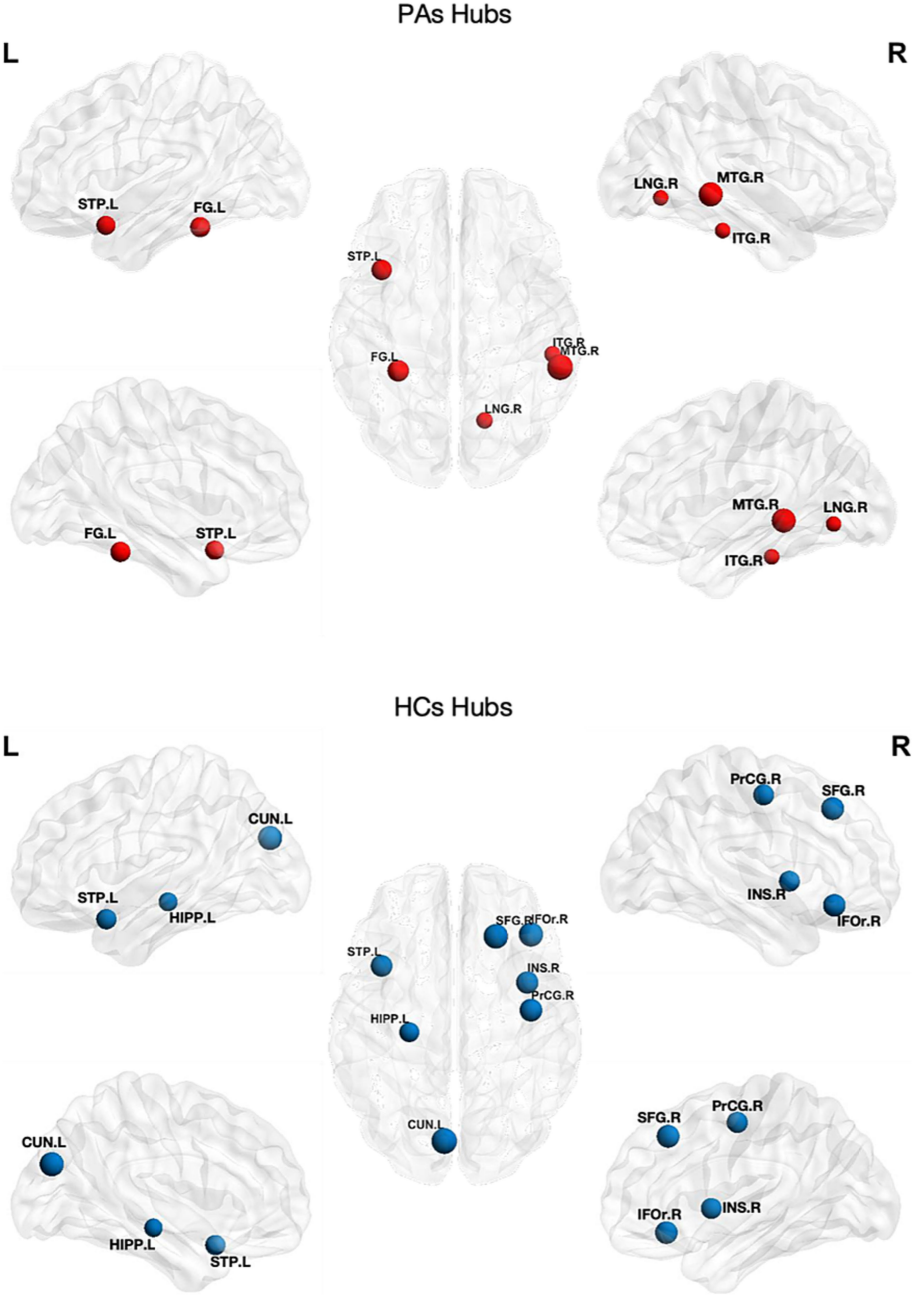
Distribution of network hubs in patients with tinnitus and HCs. The volume of the sphere represents the betweenness in the corresponding brain region. CUN, cuneus; FG, fusiform gyrus; HIPP, hippocampus; IFOr, inferior frontal gyrus, orbital part; INS, insula; ITG, inferior temporal gyrus; L, left; LNG, lingual gyrus; MTG, middle temporal gyrus; PrCG, precentral gyrus; R, right; SFG, superior frontal gyrus; STP, superior temporal pole; HCs, healthy controls; PAs, patients with tinnitus.

The network hubs were identified based on node betweenness (Figure 5). The common hub for both groups was the left STP. The hubs specific for tinnitus were detected in the left FG, right lingual gyrus (LNG), right inferior temporal gyrus (ITG), and right middle temporal gyrus (MTG). The hubs specific for HCs were detected in the left CUN, right IFOr, medial left SFG, left HIPP, right INS, and right precentral gyrus (PrCG).

### 3.6 Network robustness

**Figure 6:**
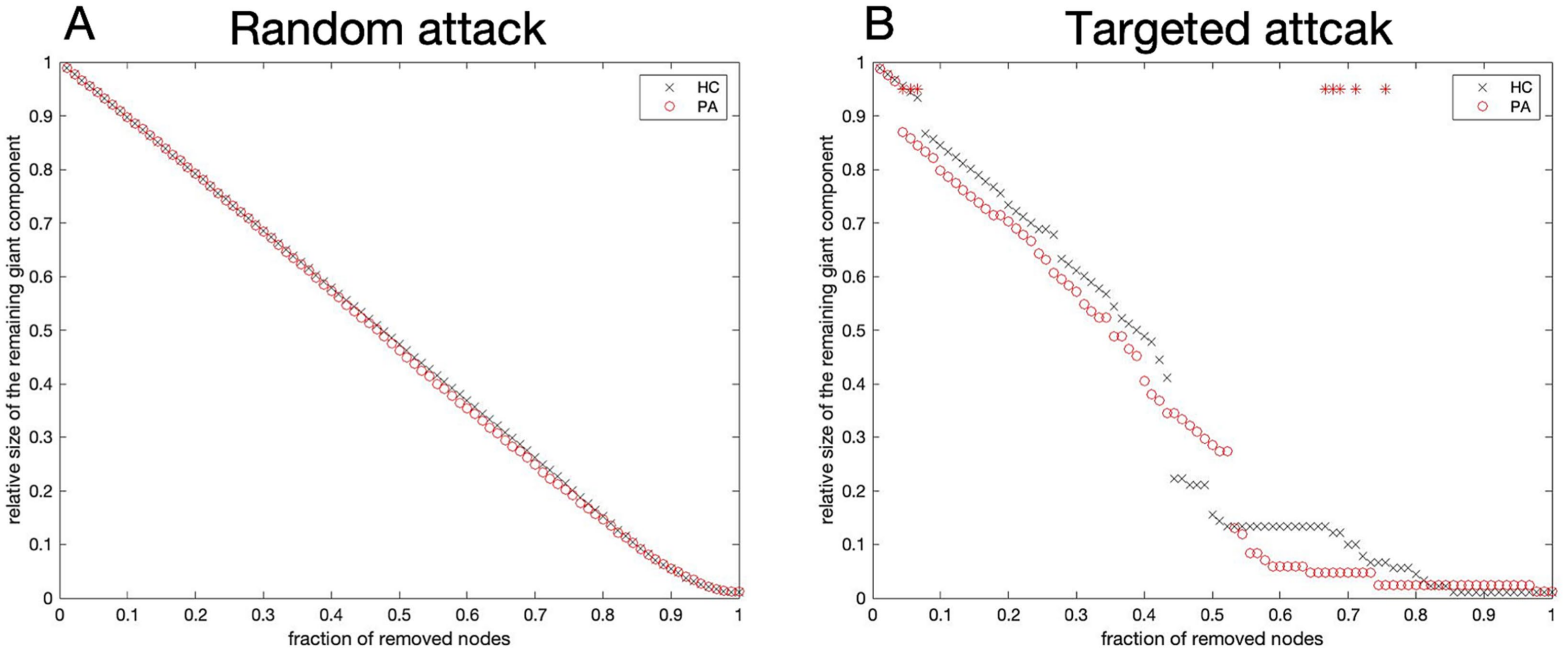
Network robustness. Variation curves of the relative size for networks resilient to random **(A)** and targeted **(B)** attacks. The resilience of both networks to targeted attacks was considerably varied in network density only in the case of removing a few nodes (red stars). The horizontal coordinate is the percentage of nodes removed; the vertical coordinate is the relative size of the network. HC, Healthy control; PA, patients with tinnitus.

Upon random attacks, the reduction in network size for both the tinnitus and HCs did not reach statistical significance, indicating no remarkable difference between the two groups in this regard (Figure 6A). The AUC results also supported this finding (*p* = 0.572). Compared with HCs, the tinnitus network was less robust to targeted attacks. Furthermore, the relative size of the identified subnetworks was considerably lower in tinnitus patients when several nodes were deleted (8/90, *p* < 0.05) (Figure 6B, red stars). The AUC results revealed that the networks of patients were as resistant to targeted attacks as those of HCs (*p* = 0.343).

### 3.7 Correlation analyses

**Figure 7:**
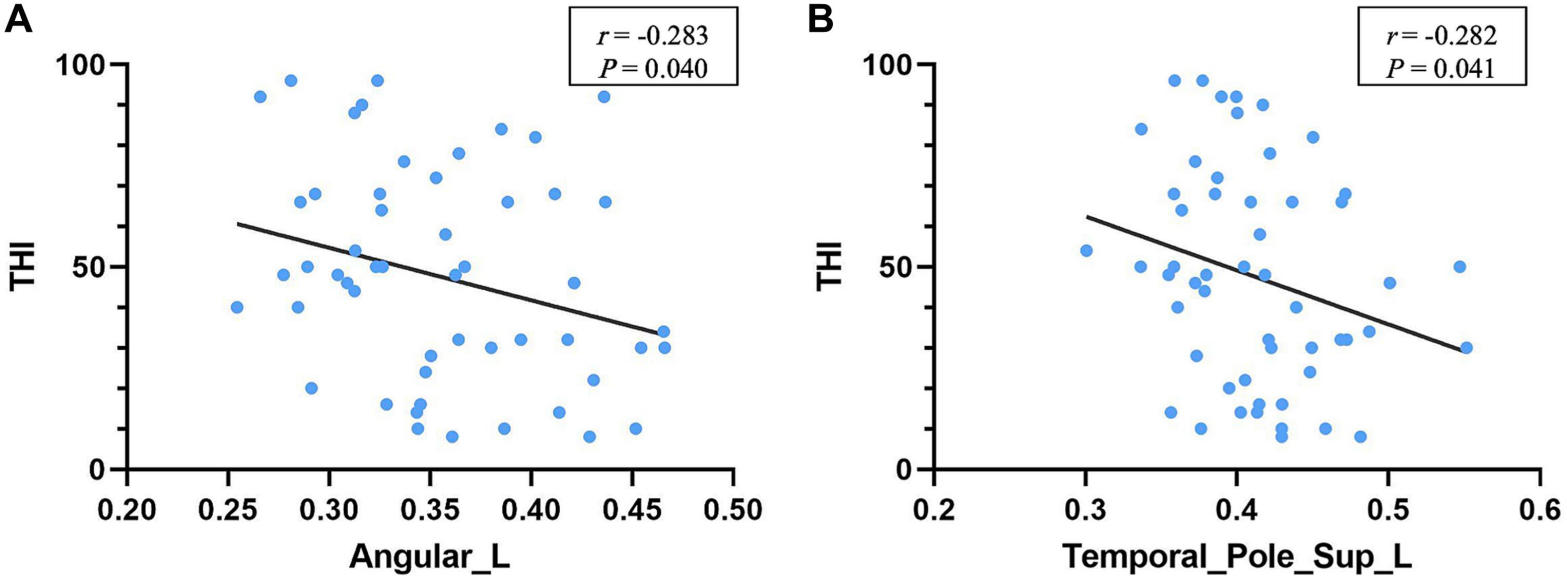
Correlation between regional GMV and THI. The higher THI score was correlated with the smaller GMV of the left angular gyrus **(A)** and left superior temporal pole **(B)** in tinnitus patients. THI, tinnitus handicap inventory.

Among the brain regions with significant differences in node analysis, the GMV values of the left ANG (*r* = −0.283, *p* = 0.040) and the left STP (*r* = −0.282, *p* = 0.041) showed a negative correlation with THI scores (Figure 7). Additionally, the GMV values of both regions were smaller in tinnitus group compared to HCs, but there was no significant difference (Table 3). However, none of regions were remarkably correlated between GMV values and tinnitus duration (*p* > 0.05).

**Table 3:** GMV comparison of the left angular gyrus and superior temporal pole between both groups.

	PAs	HCs	*P*-value
Angular_L	0.356 (0.007)	0.360 (0.006)	0.695
Temporal Pole Sup_L	0.412 (0.007)	0.419 (0.006)	0.472

HC, Healthy control; PA, patients with tinnitus.

## 4 Discussion

Structural covariation is defined as the presence of covariations in the structure or function of a brain region with other brain regions; it may reflect developmental coordination or synchronized maturation between brain regions (Alexander-Bloch et al., 2013a). To a certain extent, structural networks are thought to predict function (Seeley et al., 2009; Honey et al., 2010).

In this study, an SCN was constructed based on GMV for tinnitus patients and HCs to assess differences in brain network connectivity and topological properties between both groups. This was to determine the impact of neurological damage on tinnitus. We detected small-world properties in both groups. Patients with tinnitus exhibited increased Lp and lower Eg, resulting in substantially lower sigma. This was similar to a previous study based on rs-fMRI, which also reported higher Lp in patients with tinnitus (Mohan et al., 2016). The increased Lp might be caused by alterations and losses of hubs in patients with tinnitus, leading to a reduced ability to transfer information between long-distance regions in these patients, causing the tinnitus network to be more inclined to a regular network (Bullmore and Sporns, 2009).

Besides alterations in global properties, there were extensive alterations noted in node properties of the brain networks of patients with tinnitus, mainly the right IFOr, left PHIP, left ANG, left MOG and left OFB (*p* < 0.01). The degree of bilateral HIPP and left PHIP was remarkably reduced in patients compared with HCs, and also Hubs located in the left HIPP were lost. The HIPP and PHIP are involved in limbic system formation and associated with memory, emotion, and cognition (van Strien et al., 2009). The study by van Strien et al. (2009) found the pathophysiology impact of these regions in tinnitus-related pain and anxiety. A study based on ROI by Besteher et al. demonstrated that PHIP gray matter was reduced in tinnitus patients without psychiatric comorbidities compared with HCs (Besteher et al., 2019). Maudoux et al. (2012) also detected increased auditory–parahippocampal gyrus connectivity through connectivity mapping. Boutros et al. (2008) suggested that the PHIP region had a sensory gating function for irrelevant or redundant auditory inputs. Another functional study connected tinnitus distress and duration to the PHIP gyrus (Mohan et al., 2018).

Additionally, the ACC which belongs to the limbic system is closely related to post-traumatic stress disorder (PTSD) and executive function. Damage to the anterior cingulate gyrus causes a decrease in the ability to correct erroneous signals. The degree of the left ACC was significantly larger in patients, which indicated that patients with tinnitus might have a reduced ability of the nervous system to inhibit unnecessary sound. Furthermore, the right RLN had substantially higher clustering coefficient and local efficiencies in tinnitus patients compared to HCs. The RLN belongs to the auditory network. A previous study suggested that the hyperactivity of the RLN might lead to widespread emotional and somatosensory dysfunction in tinnitus (Job et al., 2012).

The degree of a node in a network is the number of connections it has with other nodes and this basic metric forms the degree distribution of all nodes in the network (Bullmore and Sporns, 2009). Networks with complex topological properties similar to those of the brain typically follow a scale-free power-law distributions for their degree distributions (Barabasi and Albert, 1999). In this study, the degree distributions of both networks followed an exponentially truncated power-law distribution, indicating that most nodes had a small number of connections and a few nodes had a large number of connections. The degree distribution was biased in patients with tinnitus compared with HCs, which had a normal degree distribution. This indicates that the brain network in these patients was disordered.

Assortativity is a superior description of the degree distribution of a network, which refers to the correlation between the degrees of node connectivity, and positive assortativity indicates that nodes with large degrees tend to connect to each other (Bullmore and Sporns, 2009). Our results showed that both brain networks had positive assortativity, but it was substantially larger in tinnitus patients, suggesting that the nodes with a large degree in tinnitus patients were more inclined to connect with each other and thus facilitate faster information transfer in the brain network of tinnitus patients. This was also reflected in the transitivity results, which showed higher values in tinnitus patients. Transitivity is a measure of the degree of network separation.

We further identified the ‘hubs’ of the two sets of networks based on node betweenness. Hubs typically interact with other brain regions to support network information integration and separation. The proportion of hubs is relatively small compared with that of non-hub nodes. In this study, the hubs in tinnitus patients were mostly located in the temporal and occipital lobes, while those in HCs were mostly located in the frontal, temporal, insula, and cuneiform lobes. Compared with HCs, tinnitus patients had fewer brain structural network hubs and a shifted hub distribution. Specifically, the left STG exhibited a considerably larger node betweenness in the tinnitus network, which is consistent with a previous study (Wei et al., 2020). The STG, as part of the auditory system, may be involved in the development of tinnitus. Additionally, the right LNG is crucial as a hub for tinnitus-related cognitive function and is also related to visual function.

Finally, we analyzed network resilience, which is also known as robustness. The network can be damaged by removing a small number of nodes with high degree. In this study, the robustness was similar in tinnitus patients and HCs for random attacks. However, for targeted attacks, tinnitus patients were more vulnerable. These results suggested that the SCNs of tinnitus patients had a relatively fragile topology indicating that brain networks were more easily damaged and might have more serious consequences in tinnitus patients than in HCs.

In the correlation analysis, we found a significant correlation between THI score and the left ANG as well as the left STP. The STP is considered a part of the paralimbic system while ANG is part of the attention network. The STP is associated with memory and emotion. Discomfort such as distress in patients with tinnitus may be due to the neuroelectric activity associated with tinnitus reaches the limbic system via subcortical auditory structures or auditory cortex. The ANG, which serves as a key node in the dorsal auditory pathway, is associated with memory, semantic processing and the integration of auditory stimulus, exhibiting a robust connection to the parahippocampal gyrus, a key node in the dorsal auditory pathway. A previous study demonstrated that music therapy shifts auditory hallucinations to visual attention, reducing the pain of tinnitus patients, possibly involving the ANG (Krick et al., 2017). These results suggested that tinnitus distress might be more correlated with the limbic and attentional systems rather than perceived tinnitus itself. However, no significant correlation was found between tinnitus duration and THI scores, probably because all subjects had chronic tinnitus.

This study had certain limitations. As a cross-sectional study, it cannot establish a causal link between the gray matter structural covariation network and clinical aspects of tinnitus, and further investigation requires a longitudinal and prospective design. Additionally, as tinnitus and hearing impairment have overlapping clinical symptoms, future exploration should focus on uncovering structural brain changes in tinnitus patients with hearing impairment to clarify the relationship between hearing impairment and pure tinnitus.

## 5 Conclusion

In this study, we employed graph theoretic analysis to investigate changes in the SCN of tinnitus patients and explored the correlation between topological structural properties of SCN and clinical symptoms. Our findings revealed that tinnitus patients exhibited reduced small-world properties, altered hubs, and a reduced ability to cope with targeted attacks. Additionally, the left ANG and the left STP were negatively correlated with the severity of tinnitus distress, which suggested that these areas might represent new potential targets for transcranial magnetic stimulation or electrical stimulation in the treatment of tinnitus. This study offers an objective neuroanatomical perspective on the mechanism of tinnitus, and future studies could further illuminate the pathogenesis of tinnitus through the integration of multi-scale network analysis of both structural and functional connectivity.

## Data Availability

The data analyzed in this study is subject to the following licenses/restrictions: The datasets used and/or analyzed during the current study are available from the corresponding author on reasonable request. Requests to access these datasets should be directed to Shihong Li: lishihong@fudan.edu.cn.
