# Supplementary material for: Graph analysis based on SCN reveals novel neuroanatomical targets related to tinnitus distress
Source: Front Neurosci. 2025 Jan 7;18:1417032. doi: 10.3389/fnins.2024.1417032 (PMC11747764; doi:10.3389/fnins.2024.1417032)
Supplement: Supplementary file 1 [file Data_Sheet_1.docx]

1. **Table 1. MRI data acquisition parameters**

| Protocols | Axial T2WI | Axial FLAIR | DWI | 3D-T1 MPRAGE |
| --- | --- | --- | --- | --- |
| TR (ms) | 4300 | 7000 | 1300 | 2300 |
| TE (ms) | 98 | 83 | 62 | 2.32 |
| TI (ms) | / | 2217 | / | 900 |
| B values (s/mm^2^) | / | / | 0/1000 | / |
| Slices | 20-22 | 20-22 | 20-22 | 192 |
| Dist. Factor (%) | 30 | 30 | 30 | 50 |
| Phase oversampling (%) | 10 | 0 | 0 | 0 |
| FOV read (mm) | 230 | 230 | 240 | 240 |
| FOV phase (%) | 81.3 | 81.3 | 100 | 100 |
| Slice thickness (mm) | 5 | 5 | 5 | 0.9 |
| Averages | 1 | 1 | 1 | 1 |
| Voxel size (mm^3^) | 0.6×0.6×5.0 | 0.7×0.7×5.0 | 0.6×0.6×5.0 | 0.9×0.9×0.9 |

Abbreviations: FLAIR = fluid-attenuated inversion recovery; DWI = Diffusion-weighted imaging; TR = Repetition time; TE = Echo time; TI = inversion time; FOV = Field of view.

**Supplementary figure 1. Transitivity, assortativity, and modularity comparisons and intergroup differences between patients with tinnitus and HCs.** HC, Healthy control; PA, patients with tinnitus.

1. **Differences between GMV in both groups and its correlation with THI**

The GMV in bilateral cerebellum, right Rolandic operculum (RLN), left superior temporal gyrus (STG), left paracentral lobule (PCL), and left precuneus (PCUN) were substantially higher in patients with tinnitus than in HCs (*P* <.001, uncorrected, Supplementary figure 1).

**Supplementary figure 2.** **Differences in gray matter volume between patients with tinnitus and HCs.** The results showed differences in GMV in bilateral cerebellum, right Rolandic operculum (RLN), left superior temporal gyrus (STG), left paracentral lobule (PCL), and left precuneus (PCUN), which is shown in red. HCs, Healthy controls.

1. **Abbreviations of the relevant brain regions**

AMYG amygdala

ANG angular gyrus

CALC calcarine fissure

CN caudate nucleus

ACC anterior cingulate

MCC mid-cingulate

PCC posterior cingulate

CUN cuneus

IFOp inferior frontal gyrus, opercular part

IFOr inferior frontal gyrus, orbirtal part

IFTr inferior frontal gyrus, triangular part

MedFOr medial fronal gyrus, orbital part

MFG middle frontal gyrus

MFOr middle frontal gyrus, orbital part

SFG superior frontal gyrus

MedSF superior frontal gyrus, medial part

SFOr superior frontal gyrus, orbital part

FG fusiform gyrus

HSHL heschl gyrus

HIPP hippocampus

INS insula

LNG lingual gyrus

IOG inferior occipital gyrus

MOG middle occipital gyrus

SOG superior occipital gyrus

OFB olfactory cortex

PLD lenticular nucleus, pallidum

PCL paracentral lobule

PHIP parahippocampal gyrus

IPL inferior parietal lobule

SPL superior parietal lobule

PoCG postcentral gyrus

PreCG precentral gyrus

PCUN precuneus

PUT putamen

REC gyrus rectus

RLN rolandic operculum

SMA supplementary motor area

SMG supramarginal gyrus

ITG inferior temporal gyrus

MTG middle temporal gyrus

MTP middle temporal pole

STP superior temporal pole

STG superior temporal gyrus

THL thalamus
